# Pain Outcome Determines the Sensitivity to Peripheral Opioid Antagonism of Morphine, Ibuprofen, and Their Combination in Laparotomized Mice

**DOI:** 10.3390/pharmaceutics18030392

**Published:** 2026-03-21

**Authors:** Makeya A. Hasoun, Miriam Santos-Caballero, Miguel Á. Huerta, María Robles-Funes, Amada Puerto-Moya, M. Carmen Ruiz-Cantero, Enrique J. Cobos, Rafael González-Cano

**Affiliations:** 1 Department of Pharmacology, Faculty of Medicine, University of Granada, 18016 Granada, Spain; mhasoun@correo.ugr.es (M.A.H.); msantosc@ugr.es (M.S.-C.); huerta@ugr.es (M.Á.H.); mariarobles@ugr.es (M.R.-F.); amadapm@ugr.es (A.P.-M.); 2 Institute of Neuroscience, Biomedical Research Center, University of Granada, 18016 Granada, Spain; 3 Biosanitary Research Institute ibs.GRANADA, 18012 Granada, Spain; 4 Biotechnology College, Al-Qadisiyah University, Diwaniya 58006, Iraq; 5 Department of Pharmacology, Toxicology and Therapeutic Chemistry, University of Barcelona, 08028 Barcelona, Spain; rcmaric@ugr.es; 6 Teófilo Hernando Institute for Drug Discovery, 28029 Madrid, Spain

**Keywords:** postoperative pain, laparotomy, mechanical hypersensitivity, ongoing pain, facial expressions, abdominal licking, neutrophils, opioid drugs, NSAIDs, naloxone methiodide

## Abstract

**Background/Objectives:** Postoperative pain pharmacology is complex. We investigated the sensitivity of analgesic-like effects induced by morphine, ibuprofen, and their combination to peripheral opioid antagonism in a mouse laparotomy model. **Methods**: Mechanical hypersensitivity was assessed using von Frey filaments, and ongoing pain (abdominal licking and facial expressions) was evaluated using artificial intelligence algorithms. We tested the sensitivity of the analgesic treatments to the opioid antagonist naloxone or its peripherally restricted analog, naloxone methiodide. We also tested the effects of neutrophil depletion using an anti-Ly6G antibody. Gastrointestinal transit and pupillary diameter were measured to assess non-analgesic opioid effects. **Results**: Morphine reversed all pain-related behaviors; its effect on mechanical hypersensitivity was reversed by peripheral opioid antagonism, whereas its effects on ongoing pain were not. Ibuprofen reduced mechanical hypersensitivity and facial expressions but failed to alter licking. Interestingly, the ibuprofen effect on mechanical hypersensitivity depended on peripheral opioid receptors and neutrophils at the injury site. The morphine–ibuprofen combination produced synergistic analgesia across all endpoints without enhancing opioid-induced gastrointestinal inhibition or mydriasis. Peripheral opioid antagonism reversed the effect of the combination on mechanical hypersensitivity and facial expressions but not on licking. **Conclusions:** Our results replicate the key clinical phenomena relevant to the postoperative pain context, including the potentiation of morphine analgesia by ibuprofen without the exacerbation of adverse effects. Our results suggest that drug effects on different postoperative pain measures rely on distinct neurobiological mechanisms and are not interchangeable. Therefore, the use of a battery of complementary pain endpoints in preclinical pharmacology studies is advisable.

## 1. Introduction

Postoperative pain remains a clinically significant challenge, needing a deeper understanding of its pathophysiology and pharmacology. Laparotomy, a surgical incision through the abdominal wall to gain access to the abdominal cavity, represents the initial step for open abdominal surgeries. In the United States alone, two million people undergo open abdominal surgery annually [1]. Therefore, modeling postoperative pain using laparotomy in experimental animals appears highly translationally relevant. Postoperative pain is a complex, multifaceted state that includes the development of mechanical hypersensitivity around the surgical injury and ongoing pain. Cutaneous hypersensitivity and ongoing pain are distinct entities, differing in their pharmacology and possibly in their molecular and cellular bases [2,3,4]. In animals, mechanical hypersensitivity is typically assessed using von Frey filaments [5], whereas ongoing pain has been measured by licking of the injured area or, more recently, by facial pain expressions [5,6,7]. Although both are measures of ongoing pain, they are not equivalent; while the licking response is thought to be a reflexive behavior [8], facial expressions represent an emotional response [6,7]. A thorough comparison of these measures regarding the effects of standard analgesics routinely used in postoperative pain management, such as opioids and NSAIDs, has not been previously performed.

Opioids exert both central and peripheral effects. Peripherally restricted opioid antagonists represent a valuable pharmacological tool to isolate the peripheral effects of systemic opioid agonists. This approach has been repeatedly used in rodents [9,10] and human patients [11,12]. It is generally believed that the analgesic effect of opioid drugs occurs mainly at the central level (particularly supraspinally) [11,13], although they also produce peripheral effects [14,15], in particular during inflammatory conditions [16,17,18]. Conversely, while NSAIDs do not directly bind to opioid receptors, they are hypothesized to produce part of their analgesic effects through the potentiation of the endogenous opioid system at central levels [19,20]. However, this mechanism remains largely unexplored in the periphery. Surgery triggers the recruitment of immune cells to the surgical wound. Although immune cells have a mainly pronociceptive effect [21,22], they also produce endogenous opioids [3,10], which might be susceptible to potentiation by NSAIDs. Whether the analgesic effect of NSAIDs is influenced by the opioid actions of immune cells has not been previously studied. Therefore, it is of significant interest to assess the impact of peripheral opioid antagonism on the analgesia produced not only by opioid drugs but also by NSAIDs during postoperative pain.

NSAIDs not only potentiate the analgesic effects of endogenous opioid peptides but also enhance the analgesic efficacy of opioid drugs [23]. In fact, besides being used as monotherapy, NSAIDs are frequently combined with opioids for pain relief (including postoperative pain). To the best of our knowledge, the sensitivity of the combination of an opioid and an NSAID to peripheral opioid antagonism has not been previously studied to determine whether this potentiation involves a contribution from peripheral opioid receptors.

Considering these antecedents, we studied mechanical hypersensitivity and ongoing pain (using abdominal licking and facial pain expressions as outcomes) in mice following laparotomy. We explored the effects of two gold-standard analgesics: the opioid morphine and the NSAID ibuprofen, with particular attention to the opioid-like effects induced by ibuprofen and the influence of neutrophils on its effects. We tested the sensitivity of the analgesia induced by these two drugs, administered alone or in combination, to the prototypical opioid antagonist naloxone and its peripherally restricted derivative, naloxone methiodide.

Finally, the potentiation of opioid analgesia by NSAIDs does not extend to all effects of these drugs, at least in human patients [24]. It is currently unknown whether this is also the case in experimental animals. This is relevant to determine whether mice are indeed a valid model for the study of the clinical effects of these drugs. Therefore, we also assessed the influence of ibuprofen on two distinct non-analgesic effects of morphine: inhibition of gastrointestinal transit, which is thought to be mediated peripherally [11,25,26], and changes in pupillary diameter, which are known to be mediated centrally [27,28,29].

## 2. Materials and Methods

### 2.1. Experimental Animals

Female wild-type CD-1 mice (8–11 weeks of age, weighing 25–30 g), obtained from Envigo (Horst, The Netherlands), were used for all experiments. Mice were maintained in groups of ten per colony cage within a climate-controlled facility (22 ± 2 °C). The room featured an automated 12-h light/dark cycle (lights on from 08:00 to 20:00). For environmental enrichment, each cage was equipped with a plastic tunnel and an igloo. Prior to the experimental procedures, the animals had unrestricted access to tap water and a standard laboratory diet. Group sample sizes were predetermined, relying on our prior studies that utilized identical methodological designs and experimental models [2,3]. Behavioral assessments were conducted randomly across the different phases of the estrous cycle, taking place exclusively during the light period (between 09:00 and 16:00). Each animal was subjected to a single experimental paradigm (evaluating either ongoing pain or sensory hypersensitivity). Animal welfare and care protocols adhered strictly to international regulations (European Communities Council directive 2010/63) as well as institutional guidelines. All experimental procedures received formal approval from both the Research Ethics Committee of the University of Granada (Granada, Spain) and the regional government (Junta de Andalucía) under the authorization code 24/06/2021/100A. Furthermore, this study was executed in full compliance with the Animal Research: Reporting of In Vivo Experiments (ARRIVE) guidelines.

### 2.2. Surgical Procedure

To investigate postoperative pain, all experimental subjects underwent a transverse laparotomy model. We specifically utilized female mice because the incidence of open abdominal operations in clinical settings is vastly more common in women compared to men (roughly 90% versus 10%) [1]. The surgical protocol closely followed our previously established methodology [3,4]. Anesthesia was induced using an oxygen mixture containing 3.5% isoflurane (IsoVet^®^, B. Braun, Barcelona, Spain) within an induction chamber. Throughout the surgery, anesthetic maintenance was achieved via a nose cone delivering 3% isoflurane. Animals were positioned supine, and their lower abdomens were shaved. To ensure aseptic conditions and minimize infection risks, the surgical site was sequentially disinfected using a 70% alcohol solution and a 10% povidone-iodine solution. Using surgical scissors, we executed a 1.5-cm transverse incision across the lower abdomen, oriented perpendicularly to the midline, penetrating the skin, underlying muscle layer, and peritoneum. The edges of the incision were then gently retracted to distend the injured tissues and expose the internal viscera. For wound closure, the muscular layer was sutured using simple interrupted knots, while the cutaneous layer was approximated with horizontal mattress sutures. All suturing was performed utilizing a TB15-CT 19-mm needle and Supramid^®^ 5/0 non-absorbable polyamide multifilament thread (Laboratorio Aragó, Barcelona, Spain). Sham-operated control mice were subjected to identical anesthetic induction, abdominal shaving, and aseptic preparation steps, but no incisions were made.

### 2.3. Administration of Drugs and Antibodies for In Vivo Use

In these in vivo experiments, we evaluated two primary analgesics: the NSAID ibuprofen (Sigma-Aldrich, Madrid, Spain) and the prototypical μ-opioid receptor agonist, morphine hydrochloride (Biogen, Madrid, Spain). Morphine was administered at doses ranging from 0.12 to 0.5 mg/kg, while ibuprofen was given at 8 to 32 mg/kg. We selected these specific dosage ranges based on our prior work [2], as we demonstrated that these drug doses induced dose-dependent effects in reducing mechanical hypersensitivity and in facial pain expressions. To antagonize the analgesic actions of these drugs, we utilized two compounds: naloxone hydrochloride, a centrally acting opioid antagonist, and its quaternary derivative, naloxone methiodide, which acts as a peripherally restricted antagonist (both from Sigma-Aldrich, Madrid, Spain). These were given at standard doses of 1 mg/kg and 2 mg/kg, respectively [9,30].

For all in vivo administrations, compounds were prepared in sterile physiological saline (0.9% NaCl) and delivered via subcutaneous (s.c.) injection into the interscapular area at a volume of 5 mL/kg. Control subjects received matching volumes of the saline vehicle. When assessing the combined effect of the analgesics, ibuprofen was injected right before morphine, with each compound delivered to a distinct interscapular site. Whenever opioid antagonists were required, they were administered 5 min prior to the analgesic treatments.

To ensure sufficient time for the local inflammatory response to develop, all behavioral assessments (sensory hypersensitivity, abdominal licking, and facial pain expressions) were conducted in the immediate postoperative period, 3.5 h post-surgery [2,31]. Consequently, all therapeutic agents and their combinations were injected 2.5 h following the laparotomy, yielding a 1-h pretreatment interval before testing. Finally, to rule out any behavioral alterations induced by the treatments themselves in the absence of injury, the same pharmacological protocols were evaluated in pain-free, sham-operated mice.

For the targeted depletion of infiltrating neutrophils, subjects received an intraperitoneal (i.p.) injection of an anti-Ly6G antibody (BE0075–1; Bio X Cell, Lebanon, NH, USA). Following dilution in physiological saline, this antibody was delivered at an established dose of 8 μg in a 0.2 mL volume [2,9]. Control animals were injected with either a matching volume of saline vehicle or a non-reactive isotype-matched control antibody (BE0089; Bio X Cell). All pretreatments (saline or antibodies) were systematically administered 24 h prior to laparotomy or sham procedures. Subsequent behavioral evaluations were then performed 3.5 h post-surgery.

Animals from different treatment groups were tested in a counterbalanced order.

### 2.4. Postoperative Pain Measures

Prior to the commencement of any behavioral testing, all subjects (both laparotomized and sham-operated groups) underwent a 60-min habituation phase in the testing room. To assess the onset of cutaneous hypersensitivity, we recorded mechanical withdrawal thresholds. Conversely, ongoing pain was quantified by monitoring both abdominal licking and facial pain expressions. It is important to note that independent cohorts of mice were utilized for the evoked hypersensitivity assays and the ongoing pain assessments. Furthermore, to ensure objectivity, all behavioral scoring was conducted by investigators who were strictly blinded to the experimental conditions and treatments.

#### 2.4.1. Mechanical Threshold Evaluation

Cutaneous mechanical sensitivity was assessed using the von Frey test. Animals were individually confined to opaque plastic enclosures (5 × 9 × 13 cm) positioned on an elevated wire-mesh grid. Following a 60-min habituation phase, we measured withdrawal responses using a standardized set of von Frey monofilaments (Touch-Test Sensory Evaluators; North Coast Medical Inc., Gilroy, CA, USA). These filaments, spanning target forces between 0.02 and 2 g (0.19–19.6 mN), were pressed perpendicularly against the abdominal area—roughly 2 mm adjacent to the laparotomy wound—until a slight buckling of the hair was achieved. Each stimulation lasted for 1 to 2 s and was repeated in triplicate. The probing sequence always commenced with the 0.4-g (3.92-mN) filament. A response was scored as positive if the mouse displayed rapid nocifensive actions—such as abrupt abdominal withdrawal, jumping, or licking/scratching the stimulated area—either during the filament contact or upon its immediate removal. Based on the classic up-down method, a positive reaction dictated the subsequent use of the next lower-force filament, whereas a lack of response led to the application of a stiffer filament [2]. Ultimately, the 50% mechanical withdrawal threshold was computed utilizing the Up-Down Reader software v2.0 [32]. All pharmacological and antibody treatments were assessed at 3.5 h after surgery or sham procedure, as detailed in the in vivo administration section above.

#### 2.4.2. Evaluation of Ongoing Pain: Facial Pain Expressions and Abdominal Licking

For video recording, mice were placed individually in custom-made, black-walled test compartments (50 × 120 × 60 mm) on an elevated meshed platform with a 0.5-cm^2^ grid. These compartments were arranged in a four-cubicle array, located at the edge of the platform, with the fourth wall opened and facing towards high-resolution (1440 × 1024 pixels) infrared video cameras (Kuman RPi camera, Brea, CA, USA) connected to two infrared light-emitting diodes (IR-LEDs). The test compartments were arranged to encourage the mice to look towards the visual cliff, hence facing the cameras, which were placed 25 cm from the test compartments. Each camera recorded simultaneously two mice. Cameras were controlled by raspberry pi zero single-board computers (Kubii, Lyon, France), storing video recordings on USB sticks for subsequent computer-based analysis. This custom-made device is described in detail elsewhere [33]. No experimenters were present in the testing room during evaluations. A total of 15 videos (239,901 frames), which were not used for result extraction, were selected for training purposes and manually annotated using the DeepEthogram graphical user interface (version 0.0.1) as either background (201,914 frames) or abdominal licking (37,987 frames). These annotated videos were then used to train a neural network with DeepEthogram [34] to automatically detect abdominal licking behavior in non-training videos. Using the same training video set, facial pain expressions were assessed with a convolutional neural network, following procedures previously described [2,3,33]. Briefly, DeepLabCut network (version 2.1.6.4) was trained to recognize the ears, eyes, and nose of the animals [35]. Only frames in which the mice were facing the camera (with the simultaneous detection of all these body parts with high confidence [>0.9]) were used to analyze facial pain expressions, as previously described. From these selected frames, a convolutional neural network based on Google’s InceptionV3 architecture was trained with manually annotated images classified as “pain” or “no pain”, following previously described procedures [2]. After training was completed, the software was able to examine each frame from new video recordings of laparotomized mice and assign a probability value ranging from 0 (no pain) to 1 (pain). Both the DeepEthogram-based abdominal licking classifier and the facial pain expression classifier were applied to the same video recordings, such that each evaluated video yielded two independent quantitative outputs: total abdominal licking time and the percentage of frames classified as showing facial pain.

All scripts used for Deepethogram, DeepLabCut and InceptionV3 were written in Python (v3.5). Network training and scoring of the video recordings were completed remotely on an Ubuntu Linux (version 22.04 LTS, Canonical Ltd., London, UK) computer equipped with an NVIDIA 2080Ti graphics processing unit (GPU) (NVIDIA Corporation, Santa Clara, CA, USA). All recordings of laparotomized and sham mice had a duration of 15 min. To test for the effects of drugs or antibodies on abdominal licking and facial pain expressions, mice were evaluated 3.5 h after surgery or a sham procedure (see “Administration of drugs and antibodies for in vivo use”).

### 2.5. Evaluation of Drug Effects on Gastrointestinal Transit

To evaluate gastrointestinal motility, we used a well-established charcoal meal protocol [30]. Prior to the assay, the animals underwent a 3-h fasting period during which both standard chow and water were completely restricted. Mice received 0.3 mL of 0.5% (wt/vol) activated charcoal suspension (2 g. of powder; Sigma-Aldrich Química SA, Madrid, Spain) in distilled water orally. 30 min after the administration of the charcoal meal, the animals were euthanized by cervical dislocation. The entire small intestine, spanning from the pyloric sphincter to the ileocecal valve, was meticulously excised and laid flat on a surface. The distance traveled by the leading edge of the charcoal meal was measured with a ruler. Regarding the pharmacological interventions, all investigated drugs were injected 30 min prior to the charcoal administration (60 min before euthanasia). The experimental cohorts received either the vehicle control, morphine (0.5–4 mg/kg), or ibuprofen (16–32 mg/kg). Additionally, the combination of morphine (0.5 mg/kg) and ibuprofen (16 mg/kg) was evaluated.

### 2.6. Measurement of Drug-Induced Alterations in Pupil Size

Evaluation of drug-induced changes in pupillary diameter was made as previously described [36]. Briefly, unanesthetized mice were softly restrained, and the pupillary diameter of the right eye was recorded. To minimize stress during the procedure, physical handling was strictly limited to under 10 s per measurement. The optical readings were conducted under an Olympus SZX7 stereomicroscope (Evident Corporation, Tokyo, Japan) (set at 10× magnification), which was equipped with a specialized measuring reticle in the left eyepiece (graduated at 50 divisions per 2 mm). All visual evaluations took place exactly 60 min following the subcutaneous (s.c.) administration of the respective treatments. The experimental cohorts received the identical treatment regimens described in Section 2.5.

### 2.7. Assessment of Exploratory Behavior

Exploratory activity was determined with an infrared detector (Med associated Inc., St. Albans, VT, USA) equipped with 48 infrared photocell emitters and detectors, according to a previously described method [30]. Animals were placed individually in transparent evaluation chambers (27.5 cm wide × 27.5 cm long × 20 cm high), and the distance travelled and the time spent rearing were recorded during 30 min. No experimenters were present in the testing room during the evaluation period. All evaluations took place exactly 60 min following the subcutaneous (s.c.) administration of ibuprofen (32–64 mg/kg, s.c.) or its vehicle.

### 2.8. FACS Analysis

3.5 h post-surgery, mice were euthanized via cervical dislocation to excise the abdominal incision site along with its adjacent tissue. Intact abdominal tissues from sham-operated animals served as baseline controls. The harvested samples underwent enzymatic dissociation in a solution containing 1 mg/mL collagenase IV (LS004188, Worthington, Lakewood, NJ, USA) and 0.1% DNase I (LS002007, Worthington Biochemical Corporation, Lakewood, NJ, USA). This digestion proceeded under continuous agitation at 37 °C for 60 min. To obtain a proper single-cell suspension, the digested tissues were physically triturated through a 70-μm mesh and subsequently passed into tubes fitted with 35-μm cell strainer caps.

Prior to specific staining, non-specific binding to Fc-γRII/III (CD32/CD16) was prevented by a 20-min incubation with a rat anti-CD16/32 blocking antibody (1:100 dilution; 553141, BD Biosciences, San Jose, CA, USA). The cell suspensions were then incubated on ice for 30 min with a fluorophore-conjugated antibody cocktail. This mixture included a viability dye (1:1000; 65–0865–14, Thermo Fisher Scientific, Waltham, MA, USA) alongside antibodies against the hematopoietic marker CD45 (1:200; clone 30-F11; 103108, BioLegend, San Diego, CA, USA), the myeloid lineage marker CD11b (1:100; 101227, BioLegend), and the neutrophil marker Ly6G (1:100; 127617, BioLegend). Following this primary incubation, cells were subjected to two washes using FACS buffer, consisting of phosphate-buffered saline (PBS) supplemented with 2% fetal bovine serum (FBS). Cells were subsequently fixed in 2% paraformaldehyde for 20 min and washed two additional times in the FACS buffer.

Data acquisition was performed the following day utilizing a BD FACSCanto II flow cytometer (BD Biosciences). For accurate fluorophore resolution, standard compensation beads were employed. Additionally, Fluorescence Minus One (FMO) setups were incorporated to account for baseline non-specific labeling and cellular autofluorescence. Offline data processing was conducted using FlowJo 2.0 software (Treestar, Ashland, OR, USA). Within the analysis software, sequential gating isolated the distinct immune populations: generic immune cells (CD45+ CD11b−), monocytes/macrophages (CD45+ CD11b+ Ly6G−), and infiltrating neutrophils (CD45+ CD11b+ Ly6G+).

### 2.9. Data Analysis

All statistical evaluations were done using GraphPad Prism version 8 (GraphPad Software, Boston, MA, USA). Quantitative findings are expressed as the mean ± standard error of the mean (SEM), derived from a minimum of three independent experimental replicates. The entirety of the acquired data was included in the final assessment; no outliers, experimental units, or individual animals were omitted from the analyses. To quantify the analgesic-like efficacy as a percentage, values from saline-treated sham animals were considered to represent a 100% effect (maximum pain relief), while those from saline-treated laparotomized animals were considered to represent a 0% effect (no pain relief). Statistical comparisons among multiple experimental groups were conducted via a one-way analysis of variance (ANOVA). The Student–Newman–Keuls post-test was used in all cases. A *p*-value of <0.05 was adopted as the criterion for statistical significance across all tests.

## 3. Results

### 3.1. The Peripheral Analgesic Effect of Morphine During Postoperative Pain Depends on the Pain Outcome Examined

**Figure 1:**
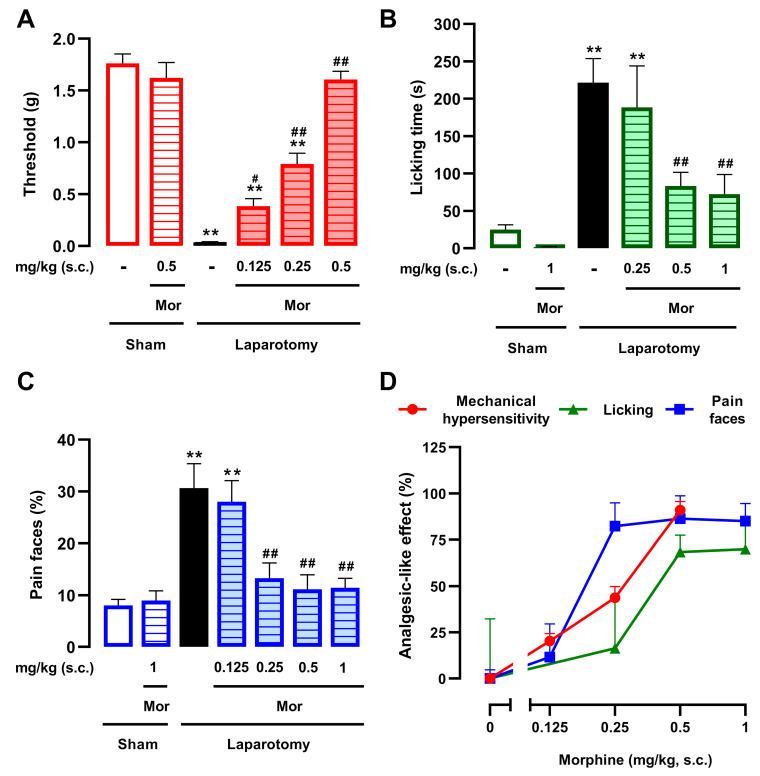
The sensitivity to the effects of morphine during postoperative pain depends on the pain outcome examined. The results represent the effects of the subcutaneous (s.c.) administration of morphine (0.125–1 mg/kg) or its solvent (saline) on (**A**) mechanical hypersensitivity (mechanical withdrawal threshold), (**B**) abdominal licking time, and (**C**) facial pain expressions in mice following a transverse laparotomy. Behavioral evaluations were performed 3.5 h after laparotomy or sham procedure. (**A**–**C**) Each bar and vertical line represents the mean ± SEM of the values obtained in 7–11 mice per group. Statistically significant differences between the values obtained in sham mice treated with vehicle (white bars) and the other experimental groups (** *p* < 0.01); and between the values obtained in laparotomized mice treated with saline (black bars) and the drug-treated groups (# *p* < 0.05; ## *p* < 0.01) (one-way ANOVA followed by Student–Newman–Keuls post hoc test). (**D**) Comparison between the analgesic-like effects of morphine (%) from data shown in panels (**A**–**C**); the values from saline-treated sham animals and saline-treated laparotomized animals were considered to represent maximum pain relief (100% effect) and zero pain relief (0% effect), respectively. Each point and vertical line represent the mean ± SEM of the values obtained in 7–11 mice per group.

We evaluated the effects of morphine, a standard opioid analgesic, on mechanical hypersensitivity and two measures of ongoing pain: abdominal licking and facial pain expressions. Laparotomized mice treated with vehicle control exhibited a substantially reduced mechanical threshold compared to uninjured animals, indicating the development of mechanical hypersensitivity (Figure 1A). Morphine (0.125–0.5 mg/kg, s.c.) produced a dose-dependent reversal of this mechanical hypersensitivity (Figure 1A). In sham-operated animals, the highest dose of morphine (0.5 mg/kg, s.c.) did not alter mechanical thresholds (Figure 1A), confirming that the drug produced analgesia without inducing nonspecific behavioral alterations of the cutaneous threshold. We then used artificial intelligence algorithms to measure abdominal licking and facial pain expressions from video recordings of our mice (see Materials and Methods for details). Vehicle-treated laparotomized animals displayed a marked increase in abdominal licking time compared to sham mice, which was significantly and dose-dependently reversed by morphine (0.25–1 mg/kg, s.c.) (Figure 1B). Regarding facial expressions, vehicle-treated laparotomized animals displayed a higher proportion of ‘pain faces’ compared to sham controls. Morphine (0.125–1 mg/kg, s.c.) also markedly reversed this measure in a dose-dependent manner (Figure 1C). In sham-operated animals, the highest dose of morphine (1 mg/kg, s.c.) did not alter abdominal licking (Figure 1B) or facial expressions (Figure 1C), confirming that the drug produced analgesia without inducing nonspecific behavioral alterations.

To facilitate the comparison of morphine’s efficacy across the various pain outcomes (mechanical hypersensitivity, abdominal licking, and facial expressions), and to more clearly illustrate the magnitude of the reversal achieved, data were represented as a percentage (%) of the analgesic-like effect (Figure 1D). Morphine sensitivity followed the order: facial expressions > mechanical hypersensitivity > abdominal licking. Notably, facial expressions were nearly fully reversed by a dose of morphine as low as 0.25 mg/kg, whereas abdominal licking consistently showed the lowest percentage of effect across the tested dose range compared to the other two measures.

**Figure 2:**
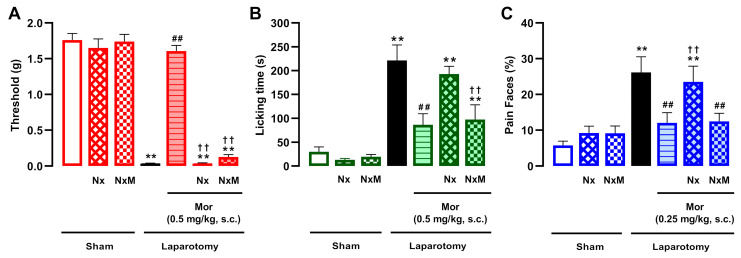
The sensitivity of morphine analgesia to peripheral opioid antagonism during postoperative pain depends on the pain outcome examined. The results represent the effects of the subcutaneous (s.c.) administration of morphine (0.5 mg/kg for mechanical hypersensitivity and licking; 0.25 mg/kg for facial expressions) combined with naloxone (Nx, 1 mg/kg, s.c.) or naloxone methiodide (NxM, 2 mg/kg, s.c.) or their vehicle (saline) on (**A**) mechanical hypersensitivity, (**B**) licking time, and (**C**) facial pain expressions in laparotomized mice. Behavioral evaluations were performed 3.5 h after laparotomy or sham procedure. (**A**–**C**) Each bar and vertical line represents the mean ± SEM of the values obtained in 7–11 mice per group. Statistically significant differences between the values obtained in sham mice treated with vehicle (white bars) and the other experimental groups (** *p* < 0.01); between the values obtained in laparotomized mice treated with saline (black bars) and the drug-treated groups (## *p* < 0.01); and between the values obtained in laparotomized mice treated with morphine alone and morphine plus opioid antagonists (†† *p* < 0.01) (one-way ANOVA followed by Student–Newman–Keuls post hoc test).

We next investigated the sensitivity of these effects to the prototypical opioid antagonist naloxone (1 mg/kg, s.c.) and its peripherally restricted derivative, naloxone methiodide (2 mg/kg, s.c.). As expected, the effects of morphine doses that markedly reversed pain-like behaviors were completely abolished by naloxone (Figure 2A–C). Interestingly, naloxone methiodide fully reversed the effect of morphine exclusively on mechanical hypersensitivity (Figure 2A), without altering its effect on abdominal licking or facial expressions (Figure 2B,C). These results suggest that while the action of morphine in cutaneous hypersensitivity is peripherally mediated, its effect on ongoing pain is not. Neither opioid antagonist altered mechanical thresholds (Figure 2A), abdominal licking (Figure 2B), or facial expressions (Figure 2C) in sham-operated animals.

### 3.2. The Effect of Ibuprofen on Postoperative Mechanical Hypersensitivity, but Not on Ongoing Pain, Is Mediated by Opioid Receptors

**Figure 3:**
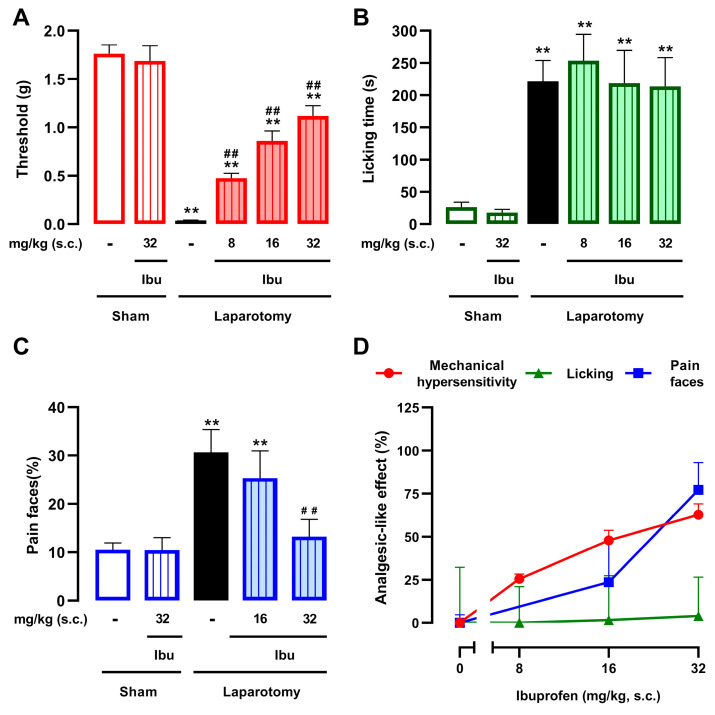
The sensitivity to the effects of ibuprofen during postoperative pain depends on the pain outcome examined. The results represent the effects of the subcutaneous (s.c.) administration of ibuprofen (8–32 mg/kg) or its solvent (saline) on (**A**) mechanical hypersensitivity (mechanical withdrawal threshold), (**B**) abdominal licking time, and (**C**) facial pain expressions in mice following a transverse laparotomy. Behavioral evaluations were performed 3.5 h after laparotomy or sham procedure. (**A**–**C**) Each bar and vertical line represents the mean ± SEM of the values obtained in 7–11 mice per group. Statistically significant differences between the values obtained in sham mice treated with vehicle (white bars) and the other experimental groups (** *p* < 0.01); and between the values obtained in laparotomized mice treated with saline (black bars) and the drug-treated groups (## *p* < 0.01) (one-way ANOVA followed by Student–Newman–Keuls post hoc test). (**D**) Comparison between the analgesic-like effects of ibuprofen (%) from data shown in panels (**A**–**C**); the values from saline-treated sham animals and saline-treated laparotomized animals were considered to represent maximum pain relief (100% effect) and zero pain relief (0% effect), respectively. Each point and vertical line represent the mean ± SEM of the values obtained in 7–11 mice per group.

We also explored the effects of the NSAID ibuprofen on mechanical hypersensitivity and ongoing pain in laparotomized animals. Ibuprofen (8–32 mg/kg, s.c.) dose-dependently attenuated cutaneous hypersensitivity; however, unlike morphine (as shown above), it failed to fully reverse this measure of cutaneous sensitivity (Figure 3A). We also evaluated the effect of ibuprofen on abdominal licking and facial pain expressions as measures of ongoing pain. Interestingly, while doses up to 32 mg/kg (s.c.)—which were effective against cutaneous hypersensitivity—failed to significantly alter abdominal licking (Figure 3B), they dose-dependently and completely reversed facial pain expressions (Figure 3C). Therefore, although both injury-site licking and facial expressions are used to assess ongoing pain, they are differentially modulated by ibuprofen. The differential efficacy of ibuprofen across pain outcomes—particularly the contrast between its effects on mechanical hypersensitivity and facial expressions, and the lack of effect on abdominal licking—is further highlighted when data are represented as a percentage (%) of the analgesic-like effect (Figure 3D).

Higher doses of ibuprofen were also considered; however, initial safety assessments in uninjured mice revealed that 64 mg/kg (s.c.)—in contrast to the innocuous 32 mg/kg dose—induced profound motor depression. This was characterized by a significant decrease in both horizontal (distance traveled) and vertical (time spent rearing) activity (Supplementary Figure S1A,B). Given that such motor impairment would interfere with the reliable assessment of pain-related behaviors, 32 mg/kg was established as the maximum feasible dose for all subsequent pharmacological evaluations. In sham-operated animals, ibuprofen 32 mg/kg (s.c.) did not alter mechanical thresholds (Figure 3A), abdominal licking time (Figure 3B), or facial expressions (Figure 3C), indicating that this drug dose did not induce nonspecific behavioral alterations that could confound the results in laparotomized animals.

**Figure 4:**
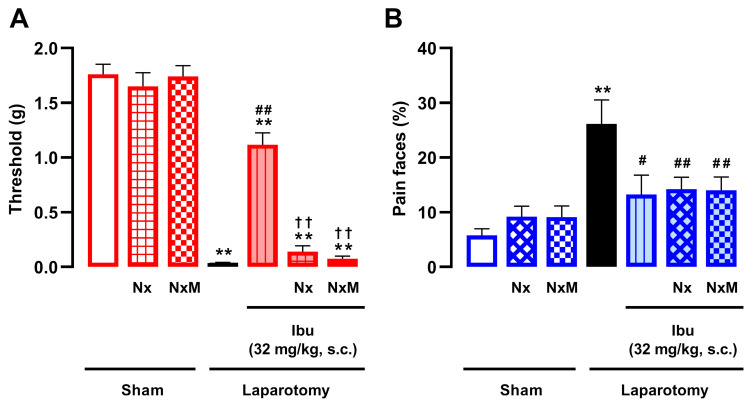
The sensitivity of ibuprofen analgesia to peripheral opioid antagonism during postoperative pain depends on the pain outcome examined. The results represent the effects of the combination of ibuprofen (32 mg/kg) with naloxone (Nx, 1 mg/kg, s.c.) or naloxone methiodide (NxM, 2 mg/kg, s.c.) or their vehicle (saline) on (**A**) mechanical hypersensitivity and (**B**) facial pain expressions in laparotomized mice. Behavioral evaluations were performed 3.5 h after laparotomy or sham procedure. (**A**,**B**) Each bar and vertical line represents the mean ± SEM of the values obtained in 7–11 mice per group. Statistically significant differences between the values obtained in sham mice treated with vehicle (white bars) and the other experimental groups (** *p* < 0.01); between the values obtained in laparotomized mice treated with saline (black bars) and the drug-treated groups (# *p* < 0.05; ## *p* < 0.01); and between the values obtained in laparotomized mice treated with ibuprofen alone and ibuprofen plus opioid antagonists (†† *p* < 0.01) (one-way ANOVA followed by Student–Newman–Keuls post hoc test).

We next assessed whether the effects of ibuprofen on mechanical hypersensitivity and facial pain expressions were sensitive to opioid antagonism by naloxone (1 mg/kg, s.c.) or its derivative, naloxone methiodide (2 mg/kg, s.c.). While the effect of ibuprofen on mechanical hypersensitivity (32 mg/kg, s.c.) was fully reversed by either opioid antagonist (Figure 4A), its effect on facial pain expressions was unaffected (Figure 4B). As mentioned previously, neither naloxone nor naloxone methiodide altered mechanical thresholds (Figure 4A) or facial expressions (Figure 4B) in sham-operated animals. Furthermore, considering that ibuprofen acts partly by potentiating endogenous opioids, we investigated whether endogenous opioid analgesia would tonically restrain postoperative pain in our model. To this end, we tested the effect of naloxone in laparotomized animals. Naloxone (1 mg/kg, s.c.) failed to alter any of the pain-related behaviors (Supplementary Figure S2A–C), ruling out a significant effect induced by opioid antagonism that could confound the interpretation of our results in either uninjured or injured animals.

Therefore, ibuprofen displayed a narrower spectrum of efficacy against postoperative pain than morphine, as it reversed mechanical hypersensitivity and facial pain expressions but failed to alter abdominal licking. Moreover, only the effect of ibuprofen on mechanical hypersensitivity appears to be mediated by peripheral opioid receptors.

### 3.3. Effect of Neutrophils on Postoperative Pain and on the Effect of Ibuprofen

**Figure 5:**
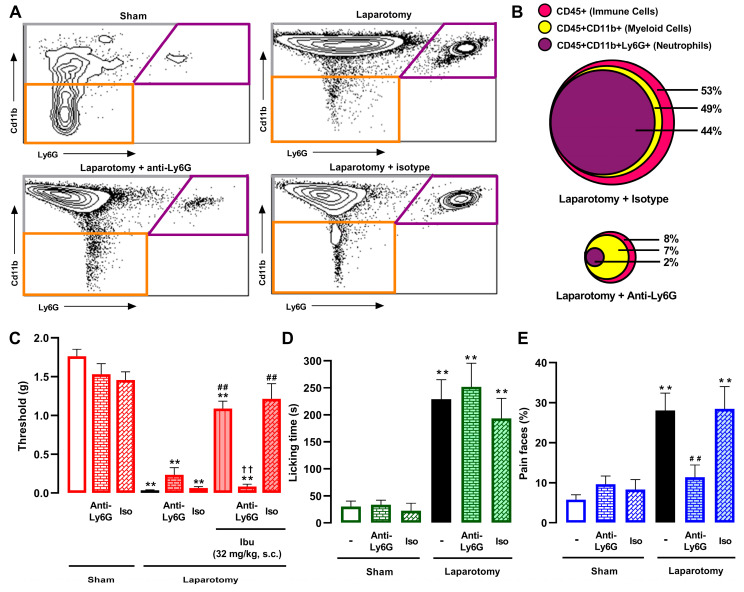
Impact of neutrophil depletion on postoperative pain outcomes and on the effect of ibuprofen in mechanical hypersensitivity. (**A**) Representative FACS diagrams, with gating from CD45+ cells, showing neutrophils (CD11b+Ly6G+, purple frame), macrophages/monocytes (CD11b+Ly6G–, grey frame) and other hematopoietic cells (CD11b–Ly6G–, orange frame) in the abdominal wall of sham-operated and laparotomized mice treated intraperitoneally (i.p.) with saline, anti-Ly6G antibody, or the isotype control (both at 8 μg). (**B**) Proportional Euler diagram showing immune cells (CD45+), myeloid cells (CD45+CD11b+) and neutrophils (CD45+CD11b+Ly6G+) in samples from laparotomized mice treated with the anti-Ly6G or the isotype control antibody (see Table 1 for the complete dataset of FACS data). (**C**) Effects of the administration of anti-Ly6G or the isotype control antibody in combination with the subcutaneous (s.c.) administration of ibuprofen (32 mg/kg) on mechanical hypersensitivity (mechanical withdrawal threshold). (**D**,**E**) Effects of the administration of anti-Ly6G or the isotype control alone on (**D**) licking time and (**E**) facial pain expressions in sham and laparotomized mice. FACS and behavioral evaluations were performed 3.5 h after laparotomy or sham procedure. (**C**–**E**) Each bar and vertical line represents the mean ± SEM of the values obtained in 7–11 mice per group. Statistically significant differences between the values obtained in sham mice (white bars) and the other experimental groups (** *p* < 0.01); between the values obtained in laparotomized mice treated with vehicle (black bars) and the drug-treated groups (## *p* < 0.01); and between the values obtained in laparotomized mice treated with ibuprofen alone and those treated with ibuprofen plus anti-Ly6G (†† *p* < 0.01) (one-way ANOVA followed by Student–Newman–Keuls post hoc test).

We performed flow cytometry (FACS) to characterize immune cell populations at the surgical injury site. As shown in Table 1, neutrophils (CD45+CD11b+Ly6G+ cells) constituted the vast majority of immune cells (CD45+) recruited to the injured abdominal wall (at 3.5 h after surgery, when all behavioral studies were performed), whereas macrophages/monocytes (CD45+CD11b+Ly6G– cells) were recruited to a lesser extent. To deplete neutrophils, we administered an anti-Ly6G antibody (8 µg, i.p.) in vivo. This treatment resulted in an almost complete reduction in neutrophils (and consequently a large portion of the total CD45+ population) in the abdominal wall of laparotomized animals, whereas the same dose of an isotype control antibody had no effect. This approach demonstrated high selectivity, as the recruitment of macrophages/monocytes was not significantly altered by the anti-Ly6G treatment, consistently accounting for approximately 5% of live cells across all laparotomized groups, regardless of the treatment received (saline, isotype control, or anti-Ly6G). Representative FACS diagrams, with gating from CD45 + cells, showing these immune cell populations in the abdominal wall of sham-operated and laparotomized mice treated intraperitoneally with saline, anti-Ly6G antibody, or the isotype control, are shown in Figure 5A. To visually synthesize the drastic alterations in immune cell populations following neutrophil depletion, we generated a proportional Euler diagram based on the FACS data (Figure 5B). Among the immune cell population (CD45+) in laparotomized mice treated with the isotype control, myeloid cells (CD11b+) and specifically neutrophils (CD11b+Ly6G+) formed the predominant subsets. Conversely, treatment with anti-Ly6G antibody resulted not only in a striking reduction in the neutrophil pool but also in a concomitant, massive contraction of the entire CD45+ population. It is worth noting that macrophages/monocytes (CD11b+Ly6G−), represented by the yellow area excluding the purple area in the Euler diagram, accounted for approximately 5% of cells in both groups, further indicating the selectivity of anti-Ly6G for neutrophil depletion.

We next assessed the impact of neutrophil depletion on the pain phenotype of laparotomized mice. Treatment with the anti-Ly6G antibody (8 µg, i.p.) failed to alter mechanical hypersensitivity (Figure 5C) or abdominal licking (Figure 5D) but prevented the increase in facial pain expressions (Figure 5E), further confirming that these three pain endpoints are distinct. Anti-Ly6G treatment did not alter mechanical thresholds, abdominal licking time, or facial expressions in sham animals (Figure 5C–E), indicating a lack of nonspecific behavioral effects. Similarly, the isotype control (8 µg, i.p.) did not influence pain-like behaviors in either sham or laparotomized animals (Figure 5C–E).

Finally, we assessed whether neutrophil depletion alters ibuprofen-induced attenuation of mechanical hypersensitivity. The anti-Ly6G antibody, but not the isotype control, fully prevented the effect of ibuprofen on mechanical hypersensitivity (32 mg/kg, s.c.) (Figure 5C). These results suggest that the opioid-mediated decrease in mechanical hypersensitivity produced by ibuprofen (described in the preceding section) requires neutrophil recruitment to the surgical site. The interaction between neutrophils and ibuprofen could not be assessed for ongoing pain, as the NSAID failed to reduce abdominal licking, and neutrophil depletion alone was sufficient to abolish facial pain expressions.

### 3.4. The Peripheral Analgesic Effects of the Combination of Morphine and Ibuprofen During Postoperative Pain Also Depend on the Pain Outcome Examined

**Figure 6:**
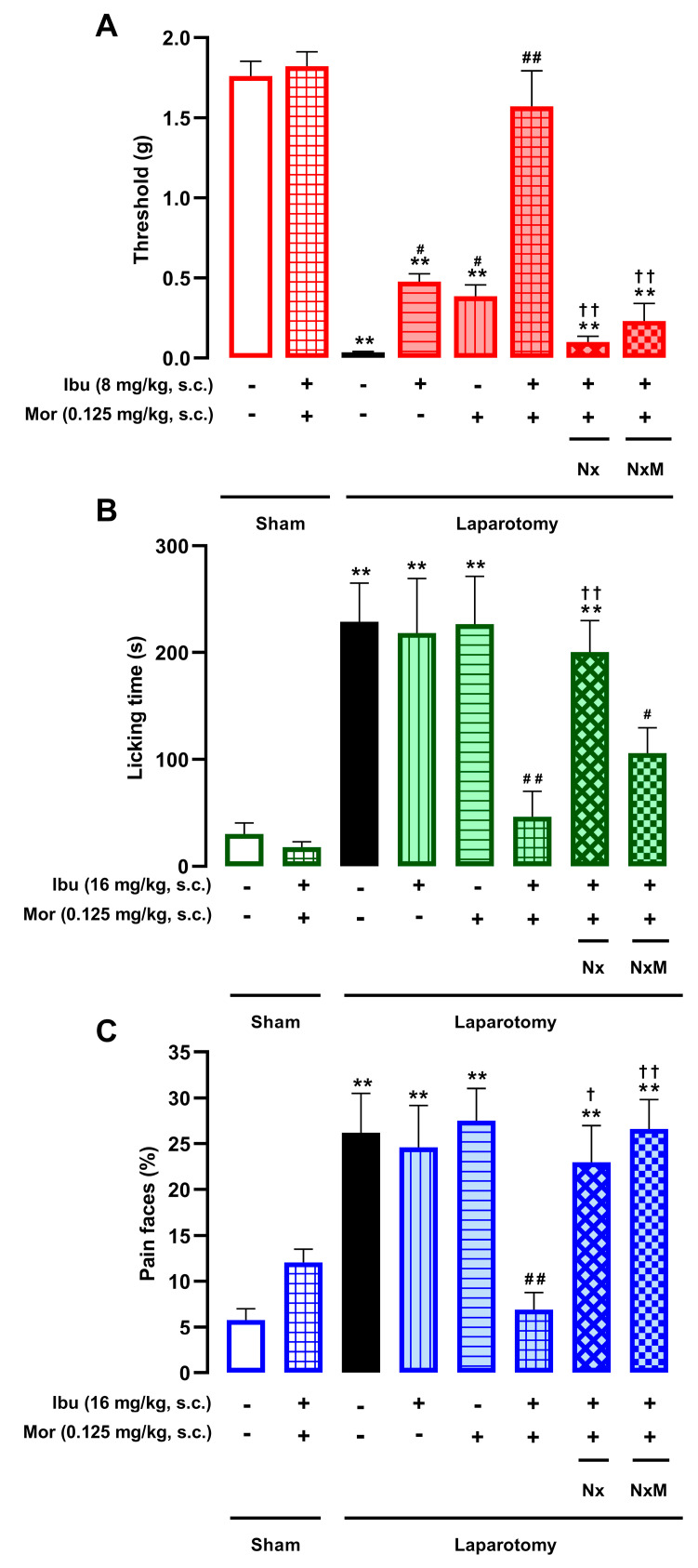
Analgesic efficacy of the morphine–ibuprofen combination across different postoperative pain outcomes and its sensitivity to peripheral opioid antagonism. The results represent the effects of the subcutaneous (s.c.) administration of sub-effective doses of morphine (0.125 mg/kg) and ibuprofen (8 mg/kg for mechanical hypersensitivity; 16 mg/kg for licking and facial expressions), administered alone or in combination, and in the presence of naloxone (Nx, 1 mg/kg, s.c.), naloxone methiodide (NxM, 2 mg/kg, s.c.), or saline on (**A**) mechanical hypersensitivity (mechanical withdrawal threshold), (**B**) abdominal licking time, and (**C**) facial pain expressions in laparotomized mice. Behavioral evaluations were performed 3.5 h after laparotomy or sham procedure. Each bar and vertical line represents the mean ± SEM of the values obtained in 7–11 mice per group. Statistically significant differences between the values obtained in sham mice treated with vehicle (white bars) and the other experimental groups (** *p* < 0.01); between the values obtained in laparotomized mice treated with saline (black bars) and the drug-treated groups (# *p* < 0.05, ## *p* < 0.01); and between the values obtained in laparotomized mice treated with the morphine–ibuprofen combination alone and those treated with the combination plus opioid antagonists († *p* < 0.05, †† *p* < 0.01) (one-way ANOVA followed by Student–Newman–Keuls post hoc test).

We evaluated the combined effects of morphine and ibuprofen on postoperative pain. Based on previous dose–response data (Figure 1 and Figure 3), we selected sub-effective s.c. doses: morphine (0.125 mg/kg) was combined with ibuprofen at 8 mg/kg for mechanical hypersensitivity and abdominal licking, or at 16 mg/kg for facial pain expressions. These combinations fully reversed mechanical hypersensitivity (Figure 6A), as well as the increase in abdominal licking (Figure 6B) and facial pain expressions (Figure 6C) in laparotomized mice, without affecting sham-operated animals. We next tested the sensitivity of these effects to the opioid antagonist naloxone (1 mg/kg, s.c.) and its peripherally restricted analog, naloxone methiodide (2 mg/kg, s.c.). Both antagonists completely abolished the antinociceptive effect of the combination on mechanical hypersensitivity (Figure 6A) and facial pain expressions (Figure 6C). In contrast, for abdominal licking, only naloxone reversed the analgesic effect, while naloxone methiodide failed to do so. These results suggest that while the morphine–ibuprofen combination is synergistic for all three outcomes, peripheral opioid receptors play a more prominent role in the relief of cutaneous hypersensitivity and facial pain expressions than in the suppression of abdominal licking.

### 3.5. Ibuprofen Does Not Increase Morphine-Induced Nonanalgesic Effects

**Figure 7:**
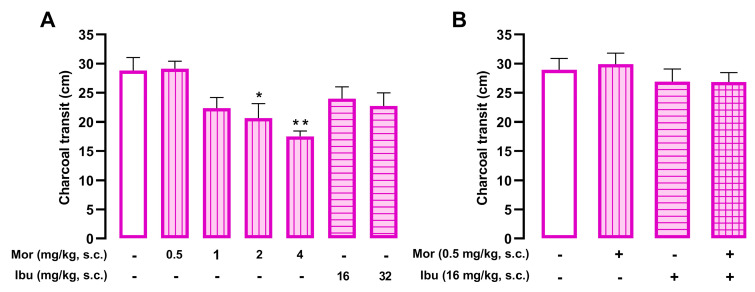
Ibuprofen does not alter the inhibition of gastrointestinal transit induced by morphine. The results represent the gastrointestinal transit distance (cm) of a charcoal suspension (0.5%) in mice treated subcutaneously (s.c.) with (**A**) different doses of morphine (0.5–4 mg/kg) or ibuprofen (16 and 32 mg/kg), and (**B**) a low dose of morphine (0.5 mg/kg) alone or in combination with ibuprofen (16 mg/kg). (**A**,**B**) Each bar and vertical line represent the mean ± SEM of the values obtained in 7–9 mice per group. (**A**) Statistically significant differences between the values obtained in vehicle-treated mice (white bars) and the morphine-treated groups (* *p* < 0.05, ** *p* < 0.01). (**B**) There were no significant differences between the values obtained in vehicle-treated mice and those treated with the low dose of morphine alone or in combination with ibuprofen (one-way ANOVA followed by Student–Newman–Keuls post hoc test).

The charcoal meal traversed approximately 30 cm through the small intestine in control animals. Morphine (0.5–4 mg/kg s.c.) dose-dependently inhibited gastrointestinal transit, whereas ibuprofen (16–32 mg/kg, s.c.) had no effect (Figure 7A). Using a strategy similar to that employed in the pain experiments, we combined sub-effective doses of morphine and ibuprofen that do not alter charcoal transit on their own. The co-administration of morphine (0.5 mg/kg, s.c.) and ibuprofen (16 mg/kg, s.c.) did not significantly decrease gastrointestinal transit compared to vehicle-treated controls (Figure 7B).

**Figure 8:**
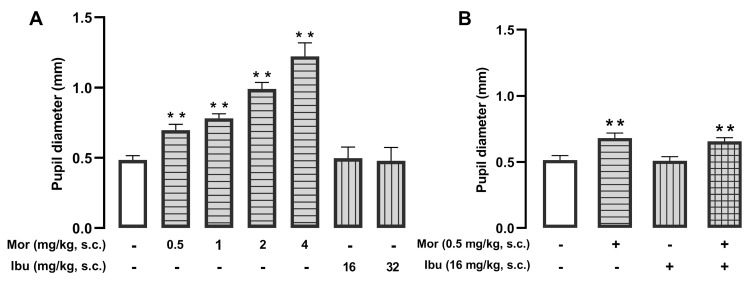
Ibuprofen does not alter morphine-induced mydriasis. The results represent the pupil diameter (mm) in mice treated subcutaneously (s.c.) with (**A**) different doses of morphine (0.5–4 mg/kg) or ibuprofen (16 and 32 mg/kg), and (**B**) a low dose of morphine (0.5 mg/kg) administered alone or in combination with ibuprofen (16 mg/kg). (**A**,**B**) Each bar and vertical line represent the mean ± SEM of the values obtained in 7–8 mice per group. Statistically significant differences between the values obtained in vehicle-treated mice (white bars) and those treated with morphine alone or the combination (** *p* < 0.01). (**B**) There were no significant differences between the values obtained in animals treated with morphine alone and those treated with the combination of morphine and ibuprofen (one-way ANOVA followed by Student–Newman–Keuls post hoc test).

We also assessed the impact of ibuprofen on another opioid side effect: mydriasis (increase in pupil size). Morphine (0.5–4 mg/kg, s.c.) induced dose-dependent mydriasis, while ibuprofen (16–32 mg/kg, s.c.) had no effect (Figure 8A). The combination of morphine (0.5 mg/kg, s.c.) and ibuprofen (16 mg/kg, s.c.) did not significantly increase pupillary diameter (Figure 8B). Therefore, despite potentiating morphine analgesia (as described above), ibuprofen did not enhance non-analgesic effects of the opioid.

## 4. Discussion

In this study, we demonstrate that the sensitivity of analgesia induced by morphine and ibuprofen (representative opioid and NSAID, respectively) to peripheral opioid antagonism in a model of postoperative pain is strictly dependent on the pain outcome assessed. We show that while the effects of morphine, ibuprofen, and their combination on mechanical hypersensitivity are predominantly mediated by peripheral opioid receptors, the relief of ongoing pain—assessed via abdominal licking and facial expressions—relies on distinct mechanisms that differ between treatments and outcomes (results summarized in Table 2). Furthermore, we identify a crucial role for neutrophils in the peripheral effect of ibuprofen in the decrease in mechanical hypersensitivity. Collectively, these findings highlight the complexity of postoperative pain pharmacology and suggest that the results of analgesic testing depend heavily on the behavioral endpoints selected.

We show that morphine was more potent in reversing facial pain expressions than mechanical hypersensitivity or abdominal licking. Although both the licking response and facial pain expressions are considered measures of ongoing pain, licking is largely a reflexive behavior [6,7], whereas facial expressions are thought to represent an emotional response [6,37]. The higher potency of morphine in reducing facial pain expressions may reflect the well-known robust effects of opioids on the emotional (affective) component of pain [38,39].

The analgesic profile of ibuprofen revealed a remarkable dissociation between pain outcomes. Unlike morphine, ibuprofen failed to inhibit abdominal licking, despite being effective against mechanical hypersensitivity and facial pain expressions. On one hand, the absence of an effect of ibuprofen on the licking response might reflect the lower efficacy of NSAIDs compared to opioids [40]. Conversely, this finding implies that a study relying solely on the licking response would have yielded a false negative for the analgesic efficacy of ibuprofen, which is one of the most commonly used analgesics worldwide.

We previously demonstrated that while the effects of morphine on cutaneous hypersensitivity can be reversed by peripheral opioid antagonism, the effect of this opioid on facial pain expressions is not [3]. Here, we extend our previous findings using abdominal licking as an additional outcome for ongoing pain and obtained identical results, as this response was also insensitive to peripheral opioid antagonism. Therefore, it appears clear that the effect of morphine on ongoing pain is mediated centrally, in contrast to its effect on mechanical hypersensitivity, which can be achieved at the periphery. Interestingly, we identified a peripheral opioid component in the effect of ibuprofen; however, this mechanism appears to be restricted to cutaneous hypersensitivity. The reduction in facial pain expressions caused by ibuprofen was insensitive to opioid antagonism, suggesting that the affective component of pain is modulated by NSAIDs through classical prostaglandin inhibition pathways independent of opioid actions. The distinct nature of mechanical hypersensitivity is relevant, given that it is currently the standard readout in rodent pain studies [7,41,42]. Here, we show that it represents a very specific pain modality; consequently, findings derived from this measure—even when using standard analgesics—are not necessarily generalizable to other outcomes, such as ongoing pain. We observed further dissociation between effects on mechanical hypersensitivity and ongoing pain, where neutrophil depletion did not alter hypersensitivity but successfully abolished facial pain expressions. This indicates that neutrophils have a pronociceptive role during postoperative pain that may go unnoticed if assessment is based solely on cutaneous sensitivity. Indeed, based on measures of cutaneous sensitivity, a recent meta-analysis of animal studies concluded that neutrophils do not contribute to postoperative pain [43]. Our results regarding the role of neutrophils in tactile hypersensitivity and facial pain expressions agree with our previous study [2]. Furthermore, they expand our findings by showing that, despite the high efficacy of neutrophil depletion on facial pain expressions, it was unable to alter the licking response. This supports the notion that both measures of ongoing pain are far from equivalent. It is worth stressing that both abdominal licking and facial pain expressions were assessed by our artificial intelligence algorithms in the same animals, which greatly facilitates the comparison between the results obtained using both endpoints.

It has been repeatedly described that NSAIDs can produce opioid-like effects at central sites by potentiating the endogenous opioid system [19,20,44]. Here, we found that the opioid-like effect of ibuprofen in the decrease in mechanical hypersensitivity was completely dependent on the presence of neutrophils at the surgical injury site. Neutrophils are known to produce endogenous opioid peptides [45,46], specifically the opioid agonist β-endorphin [3]. Therefore, our results suggest for the first time that immune-driven opioid analgesia partially accounts for the analgesic mechanism of ibuprofen during postoperative pain. Although endogenous opioids mediate some of the actions of ibuprofen, their tonic activity in the absence of the NSAID seems limited. This is evidenced by the fact that opioid receptor blockade with naloxone failed to exacerbate pain-related behaviors in vehicle-treated laparotomized mice, indicating that endogenous opioids do not exert a significant baseline inhibitory tone in this postoperative model.

Potentiation of opioid analgesia by NSAIDs is not restricted to endogenous peptides; indeed, it has long been established that NSAIDs produce synergistic analgesic effects when combined with opioids in both rodents with postoperative pain [2,23] and post-surgical patients [24,47,48]. Consistent with this, when we combined low (sub-effective) doses of morphine and ibuprofen, we observed a marked synergistic analgesic-like effect across all three pain-related endpoints (mechanical hypersensitivity, abdominal licking, and facial pain expressions). This supports the clinical utility of multimodal analgesia to achieve “opioid-sparing” effects. We also show that the effect of this combination on mechanical hypersensitivity was highly sensitive to peripheral opioid receptor antagonism. This result aligns with the effects of morphine and ibuprofen administered alone, which were already dependent on peripheral opioid activation. However, the effect of peripheral opioid antagonism on ongoing pain was more intriguing. While morphine alone acted centrally and ibuprofen alone acted via non-opioid mechanisms to reduce pain faces, their combination was fully reversed by peripheral opioid antagonism. This suggests that the simultaneous presence of both drugs creates sufficient peripheral inhibition to prevent the nociceptive discharge from triggering the supraspinal affective response. Conversely, the suppression of abdominal licking by the drug combination remained independent of peripheral opioid receptor activation, suggesting that licking represents a motor response to ongoing pain that is difficult to inhibit purely at the periphery, contrasting with the modulation of facial expressions. Again, these two responses related to ongoing pain differ in their pharmacological modulation.

Considering the robust potentiation of morphine analgesia by ibuprofen, we explored whether this NSAID would also enhance the non-analgesic effects of the opioid, selecting two distinct endpoints: inhibition of gastrointestinal transit and changes in pupillary diameter. While gastrointestinal transit inhibition is the most prominent peripheral opioid adverse effect [11,25,26,49], changes in pupillary diameter are known to be mediated centrally [27,28,50]. It is relevant to note that although morphine inhibits gastrointestinal transit in both humans [11,49] and rodents [30], it produces opposite effects on pupillary diameter in these species. While morphine induces miosis in humans [51], it causes mydriasis in both mice and rats [27,36]. Our study confirmed that morphine administration in mice led to both inhibited gastrointestinal transit and significant mydriasis. Thus, humans and rodents are not identical regarding opioid effects. We demonstrate that ibuprofen, at doses that potentiate morphine analgesia in mice with postoperative pain, does not exacerbate morphine-induced inhibition of gastrointestinal transit or mydriasis. This agrees with the known absence of modulation of opioid non-analgesic effects by NSAIDs in clinical practice [48]. Therefore, despite certain species-specific differences in opioid effects, the mouse model fully replicated the known NSAID-induced potentiation of opioid analgesia without altering non-analgesic effects—a finding that, to our knowledge, has not been previously described. It is also worth noting that while NSAIDs at therapeutic doses are generally well-tolerated, poisoning in human patients can produce several central effects, including ataxia, vertigo, dizziness, and drowsiness [52]. This clinical profile aligns with the non-specific decrease in locomotor activity observed in our animals at the highest dose of ibuprofen, which likely reflects a state of systemic malaise or mild CNS depression, further indicating a parallelism between drug effects in humans and rodents.

In conclusion, our results replicate key clinical phenomena relevant in the postoperative pain context, including the potentiation of morphine analgesia by ibuprofen without the exacerbation of adverse effects. Crucially, our findings demonstrate that different pain measures are not equivalent or interchangeable, as evidenced by the distinct pharmacological profiles and sensitivity to neutrophil depletion and peripheral opioid antagonism observed across outcomes. Consequently, to ensure a comprehensive assessment of analgesic efficacy and mechanisms, we recommend the use of a battery of complementary pain endpoints in preclinical pharmacology studies.

## Figures and Tables

**Table 1:** Immune cell populations at the surgical site following laparotomy.

Population (% of Live Cells)	Sham	Laparotomy		
		Saline	Isotype	Anti-Ly6G
CD45+ cells	3.30 ± 1.45	54.49 ± 6.31 **	52.66 ± 8.40 **	8.26 ± 2.06 ^##^
CD45+CD11b+Ly6G+ (Neutrophils)	0.69 ± 0.36	45.79 ± 4.29 **	44.25 ± 7.26 **	1.75 ± 0.68 ^##^
CD45+CD11b+Ly6G− (Macrophages/monocytes)	1.56 ± 0.88	4.24 ± 0.89 *	4.96 ± 0.76 *	5.31 ± 1.07 *

Immune cell populations at the surgical injury site were characterized by flow cytometry. Data represent the percentage (%) of each population relative to total live cells. Values are expressed as mean ± SEM (*n* = 7–10 per group). CD45: pan-leukocyte marker; CD11b: myeloid cell marker; Ly6G: neutrophil-specific marker. Statistically significant differences were assessed within each cell population: differences between the values obtained in sham mice and the other experimental groups (* *p* < 0.05; ** *p* < 0.01); and between the values obtained in laparotomized mice treated with saline or antibody (^##^ *p* < 0.01) (one-way ANOVA followed by Student–Newman–Keuls post hoc test).

**Table 2:** Summary of the analgesic effects of ibuprofen, morphine, and their combination, their sensitivity to opioid antagonism, and the impact of neutrophil depletion (anti-Ly6G) on postoperative pain outcomes.

	Ibuprofen			Morphine			Ibuprofen + Morphine			anti-Ly6G
	Sensitivity	Opioid		Sensitivity	Opioid		Sensitivity	Opioid		Sensitivity
		Nx	NxM		Nx	NxM		Nx	NxM	
von Frey	+	Yes	yes	++	yes	Yes	++++	yes	yes	–
Licking	–	–	–	++	yes	No	++++	yes	no	–
Pain Faces	++	No	no	+++	yes	No	++++	yes	yes	+

Summary of the effects of ibuprofen, morphine, their combination, and neutrophil depletion (anti-Ly6G) on mechanical hypersensitivity (von Frey), licking behavior, and facial pain expressions (Pain Faces) in laparotomized mice. Sensitivity indicates the relative magnitude of the analgesic effect observed for each treatment (+: mild; ++: moderate; +++: strong; ++++: very strong). The columns Nx and NxM indicate whether the analgesic effect was reversed (“yes”) or not (“no”) by the combination of the centrally penetrant opioid antagonist naloxone (Nx) or the peripherally restricted antagonist naloxone methiodide (NxM). A dash (–) indicates a lack of significant effect.

## Data Availability

Data presented in this study is contained within the article and Supplementary Material. Further inquiries can be directed to the corresponding author.

## Supplementary Materials

The following supporting information can be downloaded at: https://www.mdpi.com/article/10.3390/pharmaceutics18030392/s1. Figure S1. Effect of ibuprofen on the locomotor activity of uninjured mice. Figure S2. Opioid antagonism does not alter pain-like parameters in laparotomized mice.

## Abbreviations

The following abbreviations are used in this manuscript:

ANOVA	Analysis of variance
ARRIVE	Animal Research: Reporting of In Vivo Experiments
FACS	Fluorescence-activated cell sorting
FBS	Fetal bovine serum
FMO	Fluorescence minus one
GPU	Graphics processing unit
i.p.	Intraperitoneal
IR-LEDs	Infrared light-emitting diodes
NSAIDs	Non-steroidal anti-inflammatory drugs
Nx	Naloxone
NxM	Naloxone methiodide
PBS	Phosphate-buffered saline
s.c.	Subcutaneous
SEM	Standard error of the mean
