# Supplementary material for: Pain Outcome Determines the Sensitivity to Peripheral Opioid Antagonism of Morphine, Ibuprofen, and Their Combination in Laparotomized Mice
Source: Pharmaceutics. 2026 Mar 21;18(3):392. doi: 10.3390/pharmaceutics18030392 (PMC13030386; doi:10.3390/pharmaceutics18030392)
Supplement: Supplementary file 1 [file pharmaceutics-18-00392-s001.zip › pharmaceutics-4145995-supplementary.pdf]

pharmaceutics

Supplementary Materials: Pain Outcome Determines the Sensitivity to Peripheral Opioid Antagonism of Morphine, Ibuprofen, and their Combination in Laparotomized Mice

Makeya A. Hasoun[1,2,3,4*], Miriam Santos-Caballero[1,2,3*], Miguel Á. Huerta[1,2,3], María Robles-Funes[1,2,3], Amada Puerto-Moya[1,2,3], M. Carmen Ruiz-Cantero[5], Enrique J. Cobos [1,2,3,6†], Rafael González-Cano[1,2,3†]

1 Department of Pharmacology, Faculty of Medicine, University of Granada, 18016 Granada, Spain
2 Institute of Neuroscience, Biomedical Research Center, University of Granada, 18100 Armilla, Granada, Spain.
3 Biosanitary Research Institute ibs.GRANADA, 18012 Granada, Spain.
4 Biotechnology College, Al-Qadisiyah University, 58006 Diwaniya, Iraq
5 Department of Pharmacology, Toxicology and Therapeutic Chemistry, University of Barcelona, 08028 Barcelona, Spain.
6 Teófilo Hernando Institute for Drug Discovery, 28029 Madrid, Spain.
* These authors contributed equally to this work
† Correspondence: ejcobos@ugr.es, rgcano@ugr.es

 https://doi.org/10.3390/pharmaceutics18030392

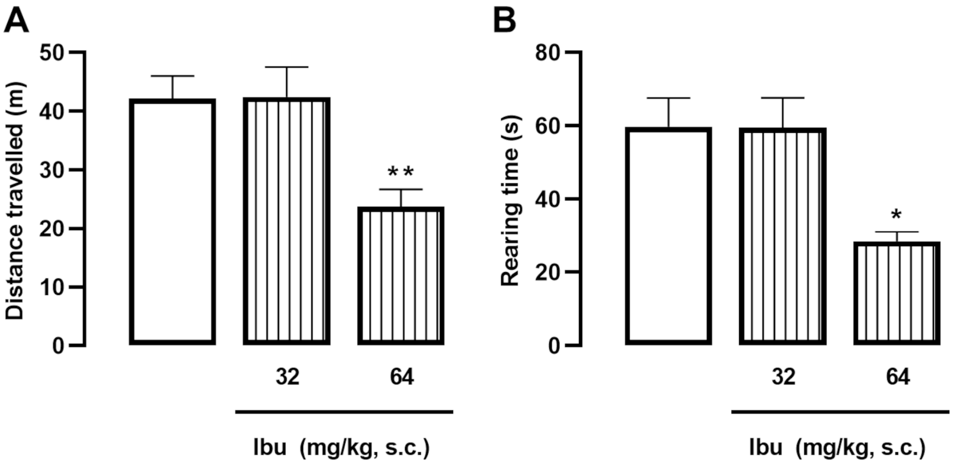

Figure S1. Effect of ibuprofen on the locomotor activity of uninjured mice. The results represent (A) the distance travelled (m) and (B) the time spent rearing (s) during a 30 min recording after the subcutaneous (s.c.) injection of ibuprofen (32-64 mg/kg) or its vehicle to uninjured mice. Each bar and vertical line represents the mean ± SEM of values obtained in 8 mice. Statistically significant differences between the values obtained in vehicle-treated mice (white bars) and animals treated with ibuprofen 64 mg/kg, s.c. (*p < 0.05; **p < 0.01) (one-way ANOVA followed by Student–Newman–Keuls post hoc test).

https://doi.org/10.3390/pharmaceutics18030392

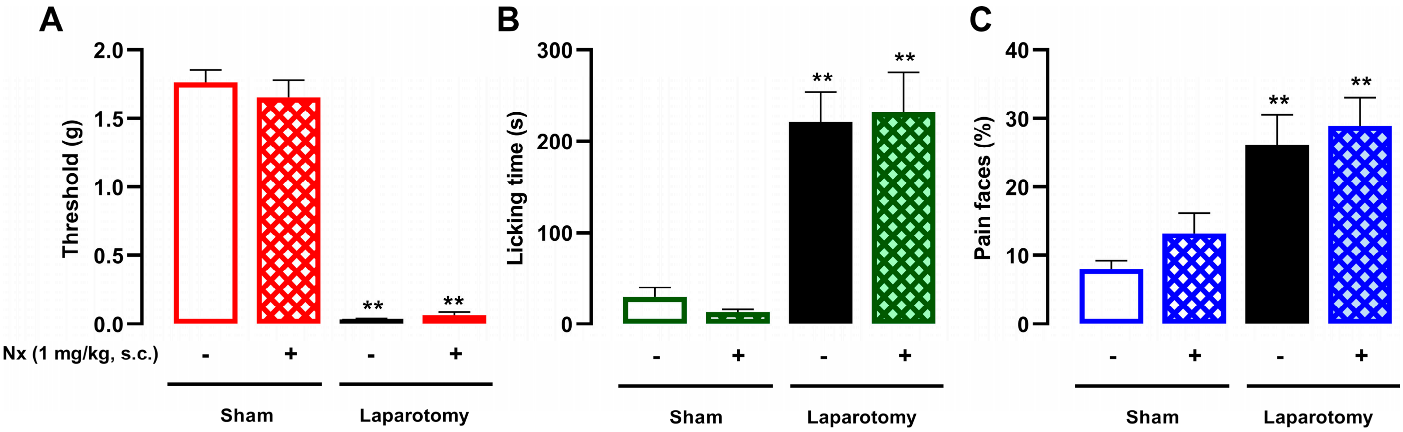

Figure S2. Opioid antagonism does not alter pain-like parameters in laparotomized mice. The results represent the effects of the subcutaneous (s.c.) administration of naloxone (1 mg/kg) or its solvent (saline) on (A) mechanical hypersensitivity (mechanical withdrawal threshold), (B) abdominal licking time, and (C) facial pain expressions in mice following a transverse laparotomy. Behavioral evaluations were performed 3.5 h after laparotomy or sham procedure. (A–C) Each bar and vertical line represents the mean ± SEM of the values obtained in 7–10 mice per group. Statistically significant differences between the values obtained in sham mice treated with vehicle (white bars) and the other experimental groups (**$p < 0.01$); naloxone administration did not significantly alter any of the parameters evaluated in either sham-operated or laparotomized mice compared to their respective saline-treated controls (one-way ANOVA followed by Student–Newman–Keuls post hoc test).

https://doi.org/10.3390/pharmaceutics18030392
